# Antigenic and sequence variability of the human respiratory syncytial virus F glycoprotein compared to related viruses in a comprehensive dataset

**DOI:** 10.1016/j.vaccine.2018.09.056

**Published:** 2018-10-29

**Authors:** Vicente Mas, Harish Nair, Harry Campbell, Jose A. Melero, Thomas C. Williams

**Affiliations:** a Centro Nacional de Microbiología and CIBER de Enfermedades Respiratorias, Instituto de Salud Carlos III, Madrid, Spain; b Usher Institute of Population Health Sciences and Informatics, University of Edinburgh, UK; c MRC Human Genetics Unit, Institute of Genetics and Molecular Medicine, University of Edinburgh, UK

**Keywords:** Respiratory syncytial virus, Genomic variability, Immunisation, F protein

## Abstract

A comprehensive analysis of sequence variation was carried out comparing the fusion (F) protein of human respiratory syncytial viruses (hRSV) from antigenic groups A and B with the prototype sequence of the A2 strain, also belonging to antigenic group A. The limited number of full bovine RSV F sequences available were included, as well as an extensive set of F sequences from the related human metapneumovirus (hMPV). The results were analysed in the context of the recently determined three dimensional F protein structures, with antigenic sites mapped to these. Although a high degree of sequence conservation in hRSV F exists, and sequence changes did not correlate with location of antigenic sites, preferential accumulation of amino acid changes in certain antigenic sites was noted. When the analysis was extended to hMPV F, a high number of changes was noticed, in agreement with the limited degree of sequence conservation. However, some conserved regions were noted, which may account for the limited number of cross-reactive monoclonal antibodies described between hRSV F and hMPV F. These results provide information about the degree of sequence and antigenic variation currently found in the F protein of circulating viruses. They highlight the importance of establishing a baseline dataset to monitor for future changes that might evolve should preventative immunological measures be made widely available.

## 1 Introduction

Human Respiratory Syncytial Virus (hRSV) is an enveloped virus of the *Orthopneumovirus* genus within the newly created *Pneumoviridae* family [1] which also includes bovine RSV (bRSV) and pneumonia virus of mice (PVM). hRSV strains are classified into two main antigenic groups - hRSV A and hRSV B- which cause seasonal epidemics in winter months and circulate worldwide. For each group a number of clades have been identified (currently 16 for hRSV A and 22 for hRSV B) [2]. hRSV has a negative stranded RNA genome which is approximately 15 kb long with 10 gene transcripts encoding 11 proteins [3], two of them being the major surface glycoproteins, namely the attachment or G glycoprotein and the fusion (F) glycoprotein.

In 2015, it was estimated that infection with hRSV resulted in 33.1 million episodes of lower respiratory infection (bronchiolitis and pneumonia) leading to 3.2 million hospitalisations and around 120,000 deaths worldwide in children younger than five years [4]. In addition, hRSV is an important cause of respiratory disease in the elderly and in immunocompromised adults, contributing to a substantial disease burden in these populations [5]. Despite such a high disease burden, no licensed hRSV vaccine is yet available. Initial attempts to create an hRSV vaccine in the 1960s were unsuccessful: a heat and formalin inactivated whole virus vaccine administered to young children did not only fail to prevent infection in the subsequent season, but led to more severe infection (enhanced disease) upon natural infection in a high percent of vaccinees and two deaths [6]. However, almost twenty prophylactic vaccine candidates and monoclonal antibodies (mAbs) are now in clinical trials, progressing from Phase I to III [7]. If, when available, they achieve widespread use, these vaccines could have a substantial effect on hRSV disease morbidity and mortality.

This new impetus in the search for a much needed hRSV vaccine originates mainly from the realisation that protection against virus infection correlates with high levels of neutralising antibodies [8], [9] which are mostly directed against one of the hRSV glycoproteins: the F glycoprotein. This glycoprotein mediates fusion of the viral and cell membranes, allowing entry of the viral ribonucleoprotein into the cell cytoplasm and thus initiation of a new infectious cycle [10].

The primary structure of the F glycoprotein consists of two segments, F1 and F2, produced by the cleavage of the precursor (F0) at Arg109 and Arg136, with the release of the intervening 27 amino acid fragment (p27). The F protein is incorporated into virus particles in a metastable conformation called prefusion, the 3-D structure of which was recently determined [11]. During membrane fusion, the F protein refolds into a highly stable conformation, denoted postfusion, the structure of which is also known [12] and which shares only some epitopes with the prefusion conformation. So far, six antigenic sites (Ø and I-V) have been identified in prefusion F, three of which are also represented in postfusion F. It has become clear in recent years that the most potent human neutralising antibodies recognise epitopes specific to the prefusion form of hRSV F [13]. Hence, most current vaccines under development, some of them already in clinical trials, rely on induction of antibody responses to epitopes of the F glycoprotein, particularly those specific to its prefusion conformation. Similarly, although palivizumab, a mAb licensed for prophylactic prevention of hRSV infections, recognises an epitope of antigenic site II shared by prefusion and postfusion hRSV F, other mAbs under development, such as MEDI8897, target epitopes of antigenic site Ø, specific to the prefusion F [14]. The F protein is also being considered as a target of small molecules under development as therapeutic agents [15].

The F glycoprotein is known to have a high level of sequence conservation among hRSV strains [16]. This level of sequence identity is also reflected in a high level of antigenic conservation. Johnson et al. [17] reported that immunisation of cotton rats with a vaccinia virus recombinant expressing the F protein from a group A virus induced cross-protective immunity not only to group A, but also group B, viruses. However, whilst the F protein may be sufficient to stimulate a cross-protective immune response between hRSV antigenic groups, there are potential scenarios in which immune selective pressures on hRSV F might act to increase F protein variation, with a possible impact on their effectiveness. The first is if a neutralising mAb targeting a specific F protein epitope were to be used so widely as to impose a strong selective pressure on hRSV F. This in turn might lead to selection for escape mutants with sequence changes in the targeted epitope. A precedent for generation of escape mutants exists both in vitro and in clinical settings. In a laboratory setting, prolonged treatment of hRSV with mAbs has been shown to select for mutants with reduced antibody binding affinities for antibodies binding to antigenic sites II, III, IV and Ø [18], [19], [20], [21], [22], [23], [24]. Clinically, cohorts of patients treated prophylactically with palivizumab or the related mAb motavizumab have been found to be infected with hRSV strains with mAb resistance-associate mutations [25], [26].

The second scenario is if an hRSV vaccine inducing F specific antibodies were to be widely used as part of vaccination campaigns. In this case, there might be a selective advantage for hRSV mutants with amino acid changes in the relevant epitopes of the F protein incorporated into the vaccine.

We therefore set out to investigate variability of F protein sequences in a comprehensive dataset of hRSV and bRSV strains and the highly related (particularly at the F structural level) human metapneumovirus (hMPV). The rationale for this approach was to identify regions of the F protein sequence which are either more conserved or more variable in the context of the prefusion hRSV F 3D structure, and its antigenic sites. Residues of higher variability may thus signal sites more susceptible to changes with immune pressure, and provide insights into the possible effectiveness of differing vaccination/prophylactic strategies.

## 2 Methods

### 2.1 Search and analysis of F protein sequences

We accessed GenBank using the search term “human Respiratory Syncytial Virus” [txid11250], “Bovine Orthopneumovirus” [txid11246] and “human Metapneumovirus” (hMPV) [txid162145] and downloaded all the available sequencing data for these organisms to the end of March 2017 (hRSV) and end of August 2017 (bRSV and hMPV). We also accessed the NIAID Virus Pathogen Database and Analysis Resource (ViPR) through the web site at http://www.viprbrc.org/ and downloaded all sequences available for “Human Respiratory Syncytial Virus” to the end of March 2017.

Using a combination of custom Python scripts and the BioPython package [27] we parsed accession numbers, collection date and country, and antigenic group (hRSV-A or B). For hRSV and hMPV, only samples with a documented collection date and country were used in subsequent analyses, to ensure that they represented natural, rather than laboratory generated, variants (due to small sample numbers this criterion was not applied to the bRSV samples). Only full-length F protein sequences without indeterminate nucleotides were included in the analyses. hRSV and bRSV sequences were aligned using MUSCLE [28] in MEGA7 [29] or MAFFT [30] to the hRSV-A A2 F protein (GenBank Accession M74568) [31]. The hMPV F protein was aligned to the A2 reference strain (which was used to elucidate the 3D structure of the pre- and post-fusion F protein) with BioEdit [32], based on the recently resolved atomic structures for these two proteins in prefusion and postfusion conformations (see Supplementary Fig. 1).

Variability in hRSV-B, bRSV and hMPV was calculated using amino acid sequences from the original nucleotide FASTA files. These amino acids, once aligned to the reference A2 strain (for bRSV, this involved truncation of the first four amino acids, which are not shared with hRSV), were compared using customised Python scripts; for each location we documented whether or not a divergent amino acid existed, and the different variants. Variability in the F protein primary structure was visualised in RStudio with the heatmap.2 function from the gplots package [33]. As A2 is a laboratory strain of RSV, analyses were repeated using the Long Strain, the oldest available hRSV-A F protein sequence (GenBank Accession JX198112) [34] dating from 1956, to ensure that our results were representative of variability with respect to a field isolate. In addition, within-group variability was calculated using the oldest available field strain for both hRSV-B (CH-18537 strain, GenBank Accession JX198143, 1962) [35] and hMPV (TN/82/5–18, GenBank Accession EU857542, 1982) [31].

### 2.2 Modelling of antigenic sites on the prefusion and postfusion F protein structure

3D structures of the prefusion and postfusion hRSV F glycoprotein were generated with PyMol [36], using PDB 4MMT [37] and 3RRR [12], respectively. Antigenic sites were defined using the six antibody epitopes (Ø and I-V) recently described by Gilman et al. [13]. Five out of these six antigenic sites were defined using a single antibody binding site; for site Ø we took the aggregate binding sites of the two antibodies used to delineate the antigenic site. Therefore the antigenic sites were defined by taking the fingerprints (5 Å pairwise distance) of the following Fab fragments: mAb D25, site Ø, (PDB: 4JHW) [11] and 5C4, site Ø, (PDB: 5 W23) [38] both bound to prefusion F; mAb MPE8, site III, bound to prefusion F (PDB: 5 V68) [39]; mAb hRSV90, site V, bound to prefusion F (PDB: 5TPN) [40]; mAb Motavizumab, site II, bound to prefusion F (PDB: 4ZYP) [41]; mAb 101F, site IV, bound to a 17 mer peptide (PDB: 2045) [12] and then modelled in prefusion F and mAb ADI14359, site I, bound to postfusion F (PDB:6APB) [42] and again modelled in prefusion F. With the exception of the site III-MPE8, since quaternary structure dependency has not been described for the binding of these antibodies to hRSVF F, to delineate each antigenic site only the residues of a single F protomer were considered. In the case of the site III, residues at 5 Å pairwise distance from Fab MPE8 of two F protomers were considered. The six antigenic sites were then mapped onto the surface structure of hRSV F folded either in the prefusion [11], [37] or the postfusion conformation [12]. The location of the six-helix bundle antigenic motif (6HB), recognised by postfusion specific mAbs [43], was placed in the postfusion F structure, based on reactivity of antibodies with F protein peptides, F protein mutants and electron microscopy.

It has been reported that the most potent neutralising mAbs bind to antigenic sites Ø, III and V, specific to (or binding preferentially to) prefusion hRSV F [11], [13], [40], [44]. Antigenic sites II and IV, shared by the prefusion and postfusion forms of hRSV F, bind mainly moderately neutralising antibodies. Antibodies binding to antigenic site I have higher affinity for postfusion than for prefusion F, and they are generally either non-neutralisers or weakly neutralising. In agreement with these observations, all the antibodies used in the study, except for ADI14339 (site I), are known to be neutralising antibodies. As the most potent neutralising antibodies bind to the prefusion form of the protein, to assess the impact of the amino acid variability on antigenicity, sequence changes were mapped to the 3D surface structure of hRSV F (A2 strain) folded in its prefusion conformation.

### 2.3 Statistical analyses of variability within antigenic sites and other regions

The probability of a mutation occurring inside a region defined as an antigenic site was tested against the probability of a mutation occurring outside these regions, for all antigenic sites and individually for each one, using a two-sided Fisher’s Exact Test implemented in R [45]. The same analyses, comparing the probability of a mutation occurring inside and outside the region, were also repeated for different regions of the F protein: signal peptide (SP, residues 1–23), 27 amino acid fragment (p27, residues 110–136), fusion peptide (FP, residues 137–154), heptad repeat A (HRA, residues 157–205), heptad repeat B (HRB, residues 485–516), transmembrane domain (TM, residues 525–549) and cytoplasmatic tail (CT, residues 549–574). The FP, HRA, HRB and TM, all present in the F1 chain, are the main elements that promote membrane fusion.

In comparisons between the A2 (or Long) hRSV F sequences and hMPV F sequences, amino acids positions in the alignment containing gaps in hRSV, and the coordinates for p27, which is not present in hMPV, were disregarded for the statistical analyses (see Supplementary Fig. 1).

### 2.4 Comparison of antigenic site II in hRSV circulating strains with laboratory mutants, bRSV and hMPV

Site II is one of the best characterised antigenic sites of hRSV F; it was initially located in the structure of prefusion and postfusion hRSV F by modelling the interactions of the mAb motavizumab (Mz) with short peptides that were co-crystallised with the Mz Fab [12], [46]. Using these coordinates, we determined the amino acid sequences found at this section of the protein, consisting of two short α-helices connected by a 6 amino acid loop (subsequent studies have shown a larger antibody footprint, once the full F protein is used to characterise the epitope) in circulating hRSV strains, and compared variability in these to that found in hRSV clinical and laboratory mutants, bRSV, and hMPV. For each amino acid in this portion of site II (residues 255–277), solvent accessible surface area (SASA) was measured, and relative solvent accessibility for each residue (Q) was obtained by normalising the SASA with standard values obtained from Gly-X-Gly tripeptides.

## 3 Results

### 3.1 Comparison of the F protein sequences from hRSV and bRSV strains and from hMPV

We downloaded 16,315 hRSV, 360 bRSV and 7627 hMPV sequences from GenBank, and 23,934 hRSV sequences from the ViPR database. We identified 1225 records with a full hRSV F protein sequence. Samples were collected between 1956 and 2016 (median 2010), and 49.7% of samples were from the United States. 791 samples were annotated as hRSV-A, and 434 as hRSV-B. Only 6 full bRSV F protein sequences were identified from GenBank, and 289 full F protein hMPV sequences.

**Fig. 1:**
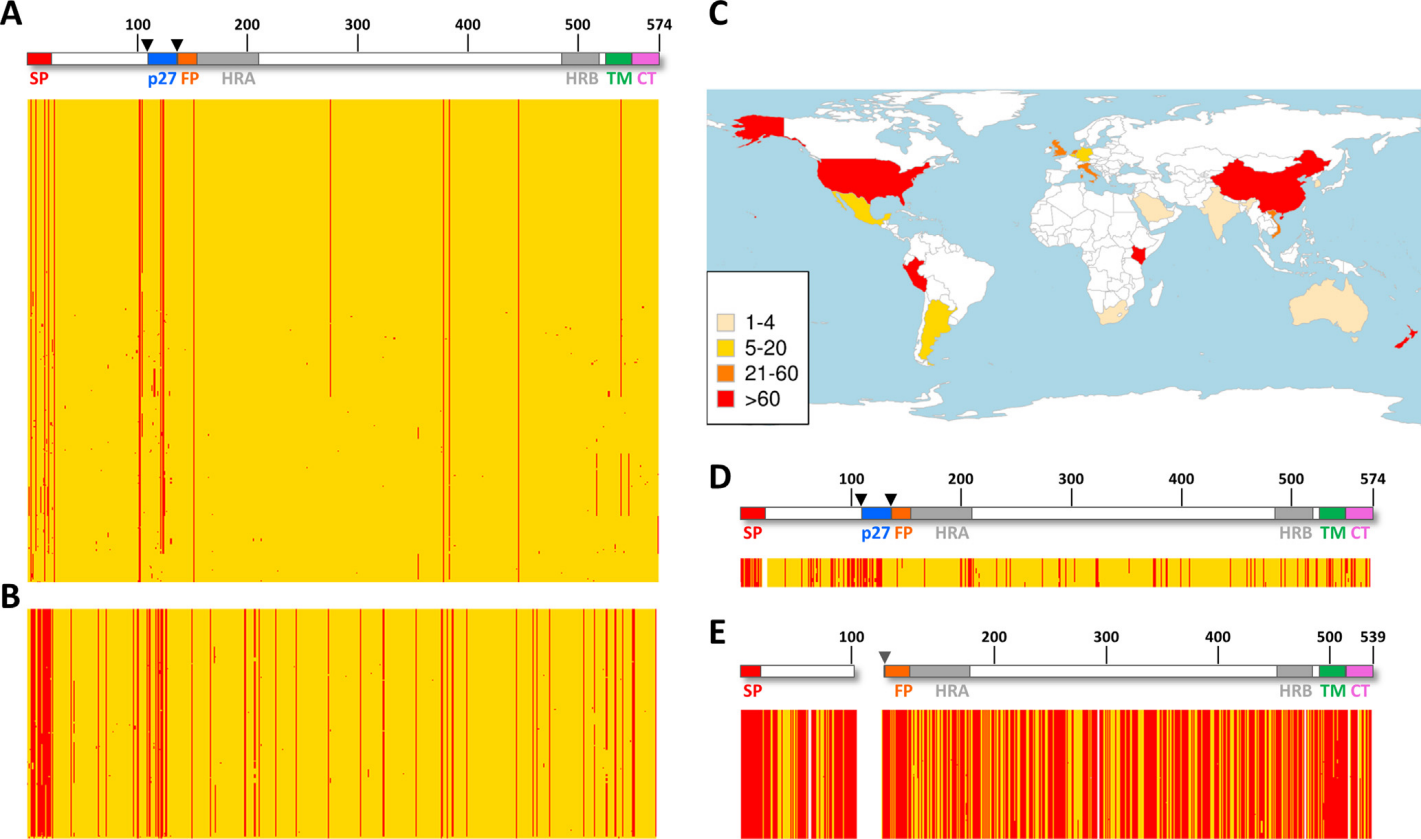
Sequence changes in F protein primary structure. (A) 791 F protein sequences classified within antigenic group A of hRSV were aligned and compared to the sequence of the hRSV A2 strain, taken as reference. Amino acid changes with the A2 strain are shown by vertical lines. A diagram of the F protein primary structure is shown at the top, denoting the following structural motifs: SP, signal peptide; p27, the 27 amino acid peptide flanked by the two cleavage sites (arrowheads); FP, fusion peptide; HRA and HRB, heptad repeats A and B, respectively; TM, transmembrane region; CT, cytoplasmic tail. (B) Representation, as in part A, of amino acid changes from 434 sequences of hRSV F antigenic group B, compared to the A2 strain. (C) World map indicating the countries from which hRSV F sequences were obtained. Colours refer to numbers of sequences from each country (inset). (D) Representation, as in part A, of the amino acid changes from 6 complete bRSV F sequences, compared to hRSV A2 strain and scheme of the F protein primary structure at the top. (E) Representation of the amino acid changes from 289 sequences of the hMPV F protein, compared to hRSV A2 strain and scheme of the hMPV F primary structure at the top. Note a gap was introduced in the hMPV F primary structure to account for the absence of p27 in this molecule.

The 791 sequences of hRSV F from antigenic group A were compared to that of reference strain A2, also belonging to antigenic group A (Fig. 1A). In agreement with previous reports, a very limited number of sequence changes were observed [16]. Some of the changes were found only in one or a few related strains whereas others were present in a large number of (or most) sequences. The changes were not evenly distributed throughout the F protein primary structure. Instead, they accumulated preferentially in the signal peptide (SP), in the 27 amino acid peptide between cleavage sites I and II (p27) and in the transmembrane domain (TM). Testing of the probability of a mutation occurring in these regions (see Supplementary Fig. 2A) showed a statistically significant increased odds ratio of this occurring at the SP (OR 17.89, 95% CI 5.79–73.72, p = 1.29E−09) and p27 (OR 13.36, 95% CI 5.07–41.45, p = 1.20E−09), but not for the TM. Of all these regions, HRA was the only one that showed a significant conservation (OR 0.27, 95% CI 0.07–0.76, p = 0.007).

As expected, the number of amino acid differences with reference to the F protein of the hRSV A2 strain (antigenic group A) increased substantially when compared with 434 sequences from hRSV antigenic group B (Fig. 1B). Accumulation of amino acid changes in SP, p27 and TM was now even more evident (with statistically significant increased odds ratios again for SP and p27, Supplementary Fig. 2B), and most changes were represented in many of the group B strains. An increased frequency of changes can also be visualised at locations corresponding to antigenic sites Ø (∼a.a. 200) and I (∼a.a. 380, see also Fig. 4). The hRSV F sequences used in Fig. 1A and B come from the countries shown in Fig. 1C. Although representing the five continents, they come from only 19 countries, and there remain many countries from which F protein sequence information is still missing.

**Fig. 2:**
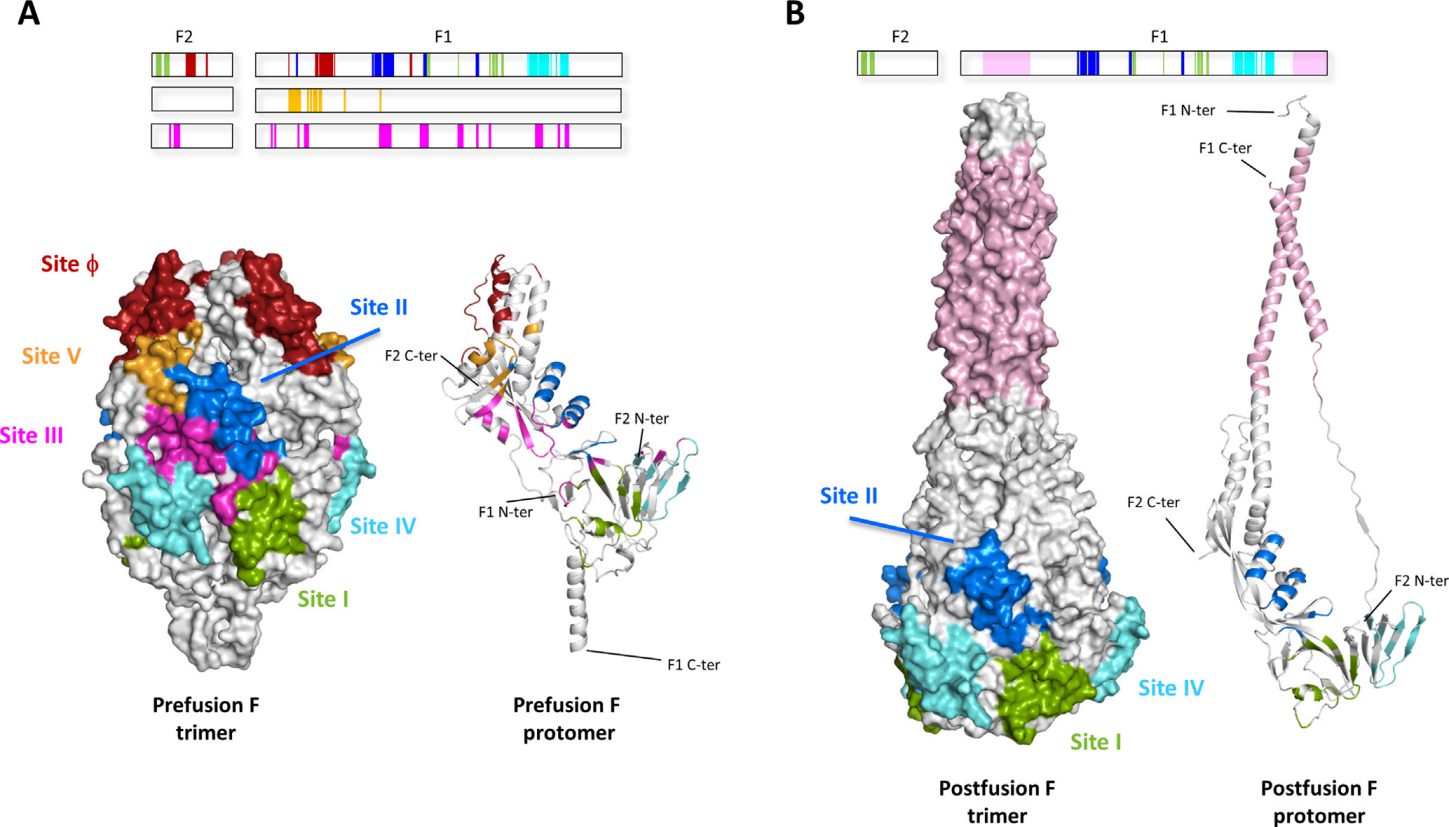
Comparison of the prefusion and postfusion antigenic structures of hRSV F. The bands at the top of Fig. 2 A and B show the primary structure of the pre-cleavage F protein with antigenic sites marked in red (site Ø), green (site I), blue (site II), purple (site III), cyan (site IV) and orange (site V). Below these to the left are surface representation of the 3D structures of hRSV F trimer folded in its prefusion (A) [37] or postfusion conformation (B) [12]. The different antigenic sites were delineated by the fingerprints of the Fab mAbs described in Materials and Methods, and are represented using the same colours as in the primary structure bands. The location of the six-helix bundle antigenic motif (6HB) is based on the reactivity of antibodies with peptides, F protein mutants, and electron microscopy. Note that only certain antigenic sites are shared by prefusion and postfusion hRSV F. To the lower right of A and B a single RSV F protomer in ribbon representation of the prefusion (A) and postfusion (B) conformation is shown, with the epitopes marked with the same colours as the bands and surface representations. (For interpretation of the references to color in this figure legend, the reader is referred to the web version of this article.)

**Fig. 3:**
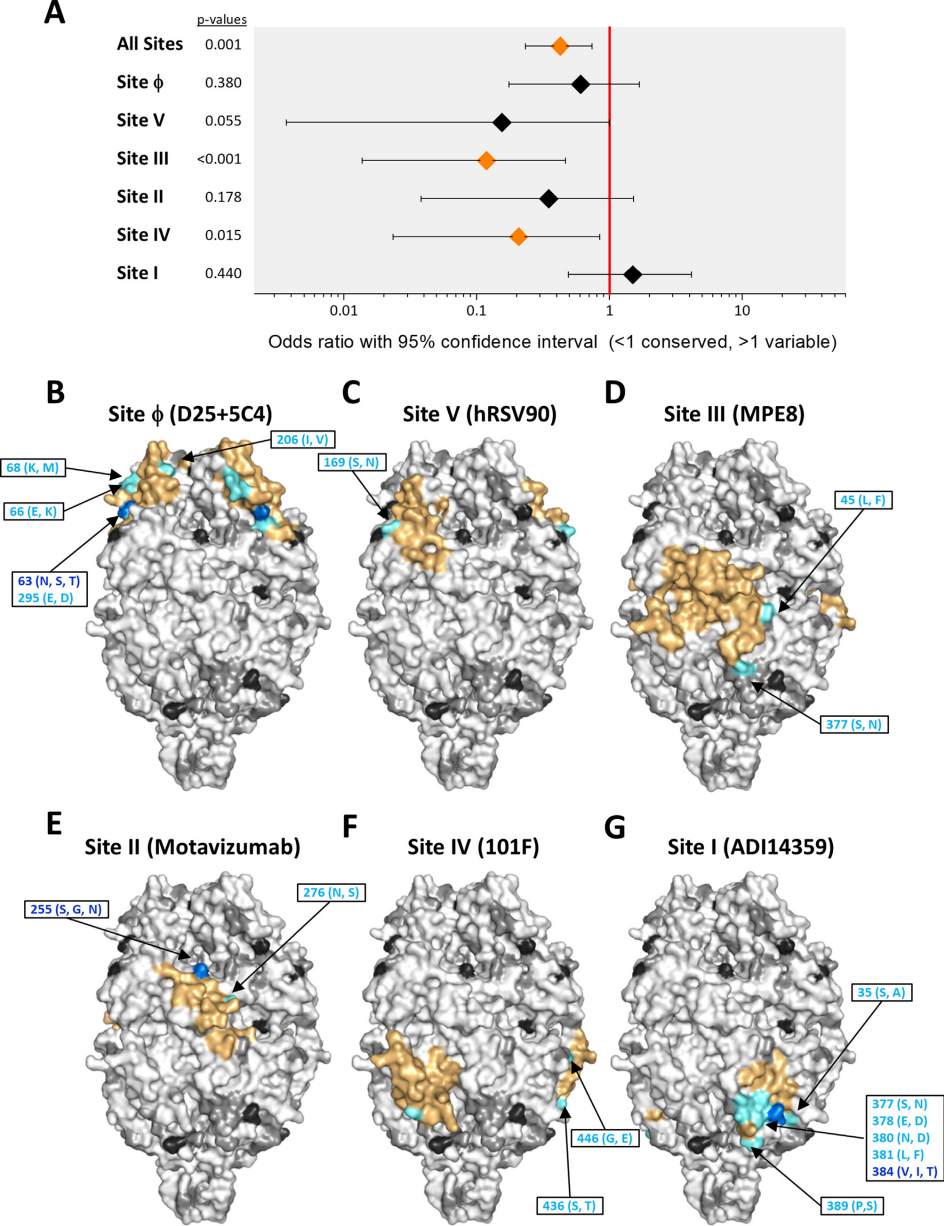
Location of amino acid differences between A2 and group A strains on the structure of prefusion hRSV F. (A) Fisher’s exact tests are shown for the probability of a mutation occurring inside or outside an antigenic site. For each antigenic site and for all antigenic sites grouped together (“All sites”), data are presented as an odds ratio with a 95% confidence interval. Significantly (p < 0.05) increased or reduced values are displayed in blue and orange, respectively, with non-significant odds ratios shown in black. (B–G) The surface of the prefusion F trimer is shown in each panel with the indicated antigenic site delineated in color as identified in Fig. 2. Within each antigenic the amino acids are colour based on the number of changes in a given residue: no variants in orange, 1 variant in cyan, 2 variants in blue and 3 variants in red. Amino acid changes included in each antigenic site are denoted in the different panels. When the number of amino acid changes prevented showing them individually, the changes were included in a box, as for instance in antigenic site I (panel G). The changes are listed in Supplementary Table S1. Amino acid outside the antigenic site are shown in increasing intensities of grey and black, where white indicates sequence conservation. (For interpretation of the references to color in this figure legend, the reader is referred to the web version of this article.)

**Fig. 4:**
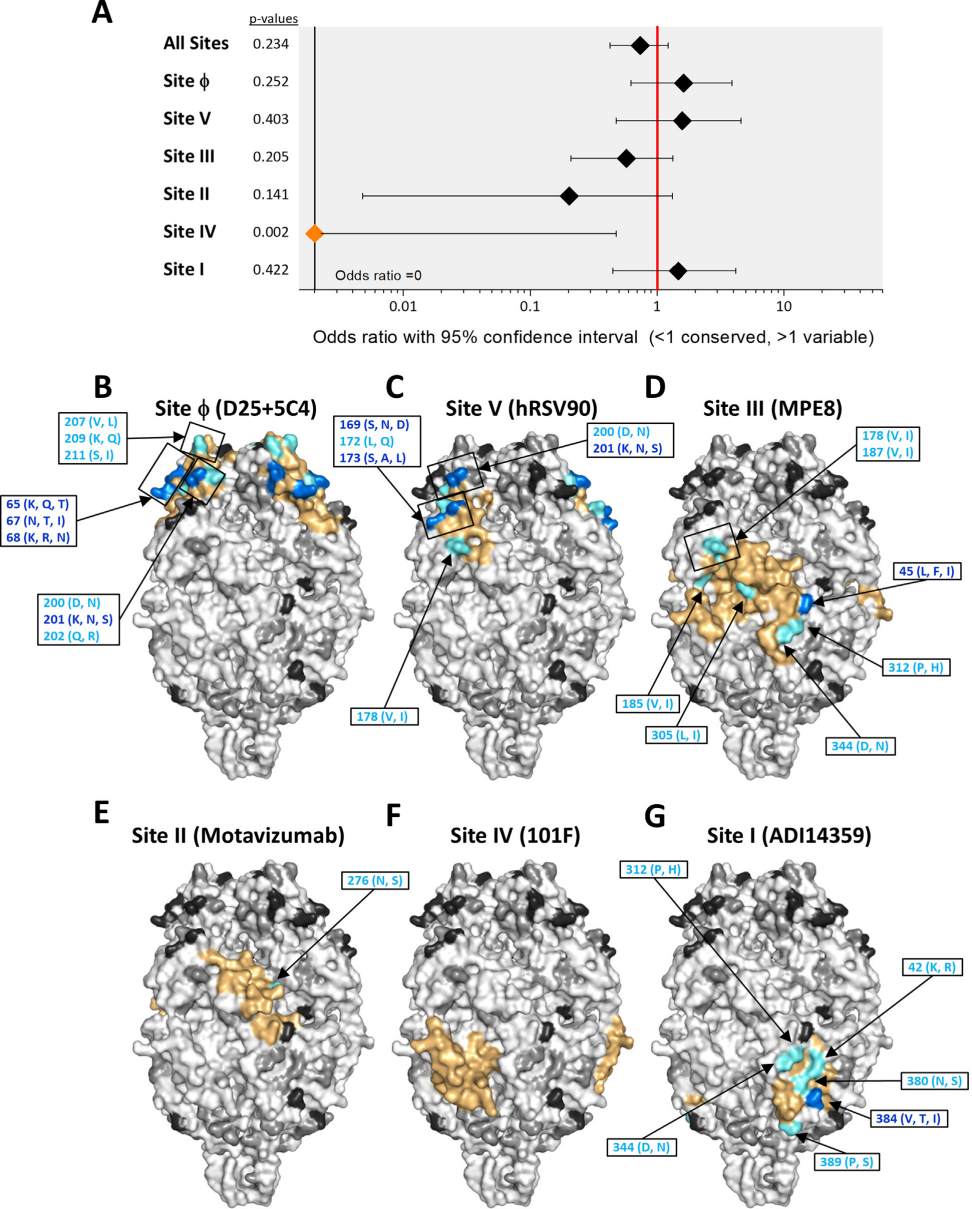
Location of amino acid differences between A2 and group B strains on the structure of prefusion hRSV F. Structures and changes are shown as in Fig. 3. The changes are listed in Supplementary Table S2.

Only six complete bRSV F protein sequences were available. Nevertheless, when compared with the A2 sequence, a significant number of sequence changes are observed, most of them shared by the available bRSV samples (Fig. 1D). These changes accumulate preferentially in the SP, p27 and TM regions of the F primary structure, again with significant increases in the odds ratios for SP and p27 (Supplementary Fig. 2C). Finally, the number of amino acid changes for hMPV F compared with the hRSV A2 strain was very high, in agreement with only 33% approximate sequence identity of hMPV F and hRSV F [47]. Sequence differences between hMPV F and hRSV F were distributed throughout the entire primary structure, although some patches of conserved sequences were discernible in the F1 and F2 chains.

Re-analyses of the same data using F Long as the reference protein showed equivalent results for groups analysed (Supplementary Fig. 3). Within-hRSV group B variability revealed similar patterns to that shown within hRSV group A (Supplementary Fig. 4A). Sequence changes accumulated preferentially in the SP region and p27, although HRA is not conserved as in the case of group A. Analyses of within hMPV F protein variability showed significant variability only in the TM (Supplementary Fig. 4B).

### 3.2 Location of amino acid sequence changes in the 3D structure of the prefusion F protein and association with antigenic sites

Fig. 2A depicts the six major antigenic sites on the surface of the F trimer (left) and in a ribbon representation of an F2/F1 monomer (right) both folded in the prefusion [11], [37] conformation. Fig. 2B shows the equivalent location of antigenic sites I, II and IV (shared by prefusion and postfusion F) on postfusion F, and additionally the site recognised by mAbs specific to the six-helix bundle (6HB) motif characteristic of postfusion F.

**Fig. 5:**
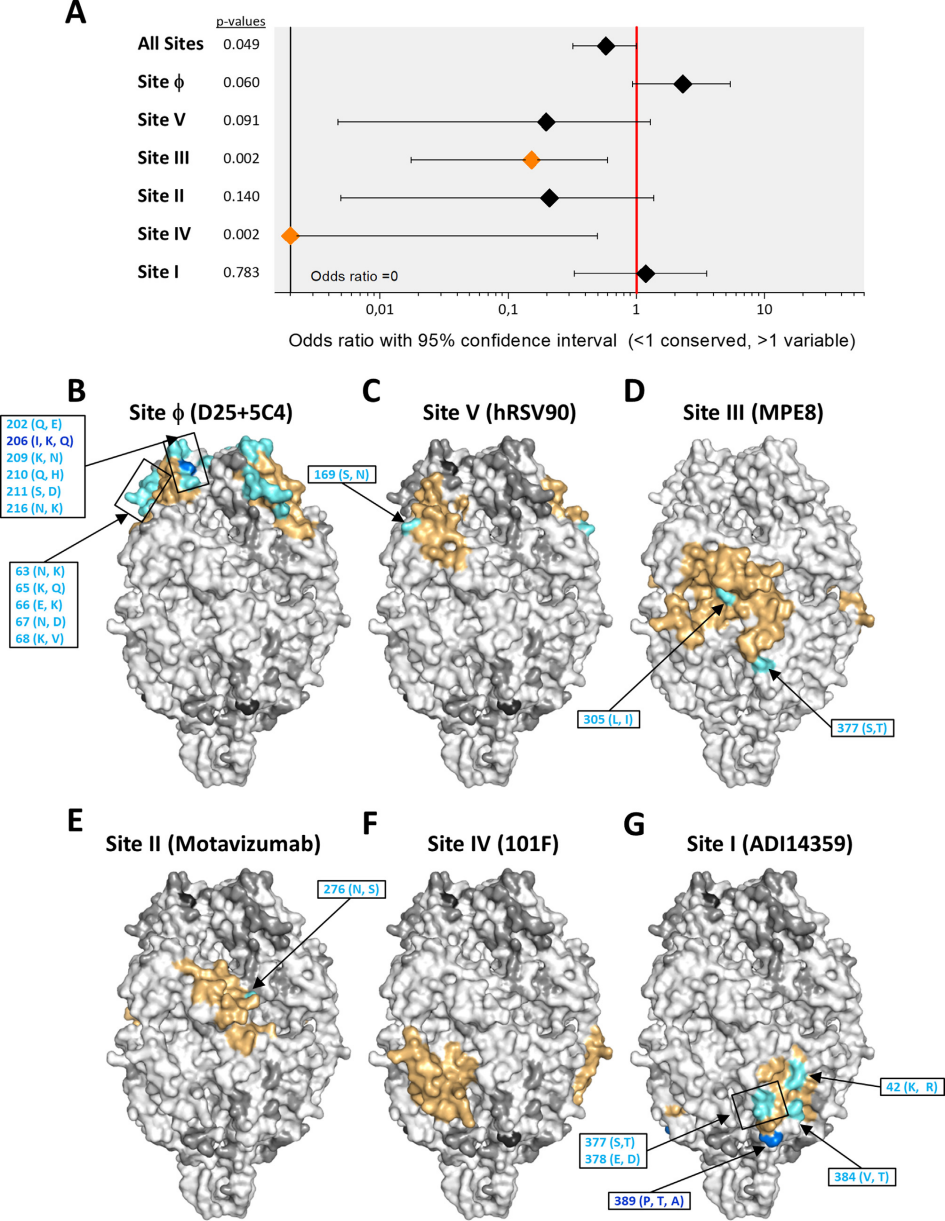
Location of amino acid differences between A2 and bRSV strains on the structure of prefusion hRSV F. Structures and changes are shown as in Fig. 3. The changes are listed in Supplementary Table S3.

**Fig. 6:**
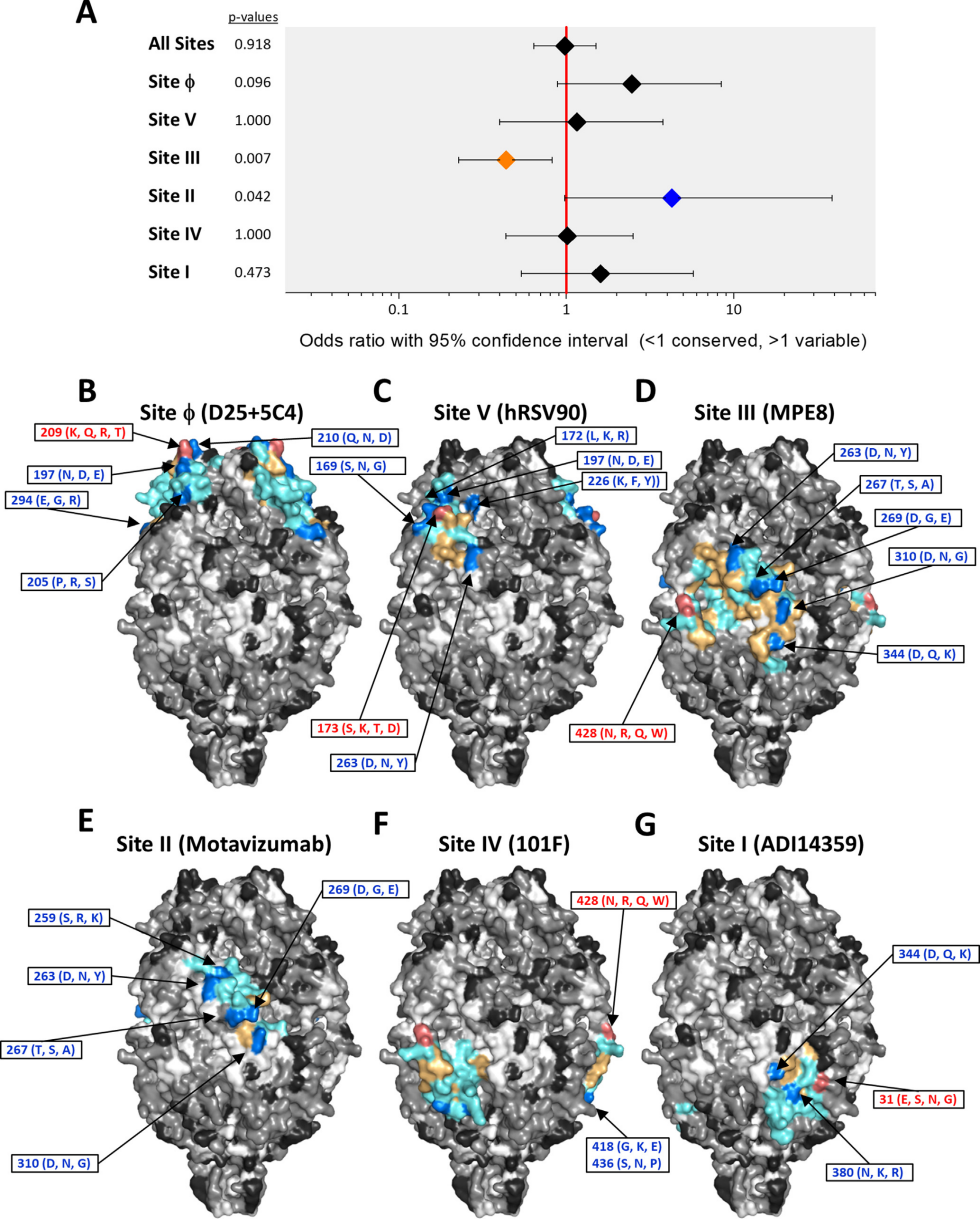
Location of amino acid differences between A2 and hMPV strains on the structure of prefusion hRSV F. Structures and changes are shown as in Fig. 3. The changes are listed in Supplementary Table S4.

To assess the impact of the amino acid variability described above on antigenicity, these changes were mapped to the 3D surface structure of hRSV F (A2 strain) folded in its prefusion conformation (Fig. 3, Fig. 4, Fig. 5, Fig. 6). Changes within hRSV group A (listed in Supplementary Table 1) are shown in the prefusion F trimer of the panels of Fig. 3B–G, where each of the six antigenic sites noted previously have been individually delineated to facilitate visualisation (coloured surface). Odds ratios with associated confidence intervals and p-values for all the antigenic site are shown in Fig. 3A. Overall, there was a significant decrease in the probability of a mutation occurring within or outside an antigenic site for all 6 sites (OR 0.42, 95% CI 0.23–0.74, p = 0.001). This is in agreement with the high level of antigenic site conservation within group A viruses noted previously with monoclonal and polyclonal antibodies [19], [48], [49]. Within antigenic sites, the limited sequence variation was mostly restricted to certain residues of antigenic sites Ø and I. Sites III (OR 0.12, 95% CI 0.01–0.47, p = 1.55E−5) and IV (OR 0.21, 95% CI 0.02–0.84, p = 0.015) showed significant conservation compared to non-antigenic site regions.

The location of the sequence changes in group B hRSV F compared with the A2 strain are shown in Fig. 4 and listed in Supplementary Table 2. For hRSV B there was no difference in the probability of a mutation occurring within or outside an antigenic site for all 6 antigenic sites (OR 0.73, 95% CI 0.43–1.22, p = 0.234) when A2 was used as the reference. Site Ø accumulated the highest proportion of mutations of the antigenic sites, in agreement with the group specificity of certain mAbs binding to this site [38]. Sites V and I also showed an increased proportion of changes, compared to other antigenic sites, although again this was not significantly increased with respect to non-antigenic sites. As with within- hRSV A variability, Site IV showed significant conservation compared to non-antigenic sites (OR 0.00, 95% CI 0.00–0.48, p = 0.002). However, unlike for the hRSV A sequences, site III showed no significant conservation, although it was, together with site II, the most conserved antigenic site after site IV.

The changes of the A2 strain compared with the limited number of bRSV F sequences are shown in Fig. 5 and listed in Supplementary Table 3. When compared with the amino acid differences between hRSV F of group B strains and the A2 strain, it seems that bRSV F may show even fewer differences, but this is likely a consequence of the small number of bRSV F sequences available. However, their distribution on the prefusion F structure is similar to that for RSV A and B, with antigenic site Ø accumulating the most differences (OR 2.30, 95% CI 0.94–5.41, p = 0.06) and sites III and IV showing relative conservation. Overall, the similarities between bRSV and hRSV are in agreement with the high level of cross-reactivity between bRSV F and hRSV F observed with murine and bovine mAbs [50] as well as with certain human mAbs [44].

Finally, the number of sequence changes between hRSV F A2 strain and hMPV F sequences were very high, and distributed throughout most of the prefusion F surface, including residues in the six antigenic sites (Fig. 6 and Supplementary Table 4); as with hRSV B and bRSV there was no difference in the probability of a mutation occurring within or outside an antigenic site region (OR 0.98, 95% CI 0.64–1.50, p = 0.918). However, some patches of conserved residues were noted in antigenic sites III, which showed relative conservation compared to other non-antigenic sites (OR 0.44, 95% CI 0.23–0.83, p = 0.007) which may account for the observed cross-reactivity between hRSV F and hMPV F with a site III specific mAb (MPE8) [44]. None of the mutations which have been shown to reduce MPE8 binding (R49D,T50A L305R, G307R and D310A) [44] were identified in either the hRSV or hMPV strains. Although site IV did not show significant conservation compared to non-antigenic sites, it had the lowest proportion of changes after site III, which may again explain the cross-reactivity of two mAbs binding to this site for hRSV and hMPV, one of which is 101F, used to define this site in our study [51], [52]. The two most variable antigenic sites when comparing the A2 reference with the hMPV F sequences were site II and to a more minor extent site Ø. It is possible that the variability seen in site II, not observed in the circulating hRSV or bRSV sequences, indicates the potential for this region of the protein to incorporate amino acid changes, some of which are also observed in the hRSV site II escape mutants discussed below.

As in the case of F regions previously commented, re-analyses of variability within the antigenic sites using F Long as the reference protein showed equivalent results for all groups and species (Supplementary Fig. 3). In addition, within hRSV B analyses showed that, as when A2 or F Long was used as a reference, (i) there was no difference in the probability of a mutation occurring within or outside an antigenic site for all 6 antigenic sites, (ii) site Ø, V and I accumulated the highest proportion of mutations of the antigenic sites, and (iii) site IV again showed significant conservation compared to non-antigenic sites (Supplementary Fig. 4A). Finally, analysis of within hMPV variability showed that the antigenic site with the highest variability was site V (OR 4.7, 95% CI 1.62–13.45, p = 0.002) rather than site Ø (Supplementary Fig. 4B). This observation is in agreement with the hypothesis that site Ø accessibility to antibodies is limited due to a dense glycan shield at the apex of the hMPV F protein [53], so that immune pressure resulting in amino acid variability may be reflected to a greater extent in other antigenic sites, such as site V or site II.

### 3.3 Antigenic site II

**Fig. 7:**
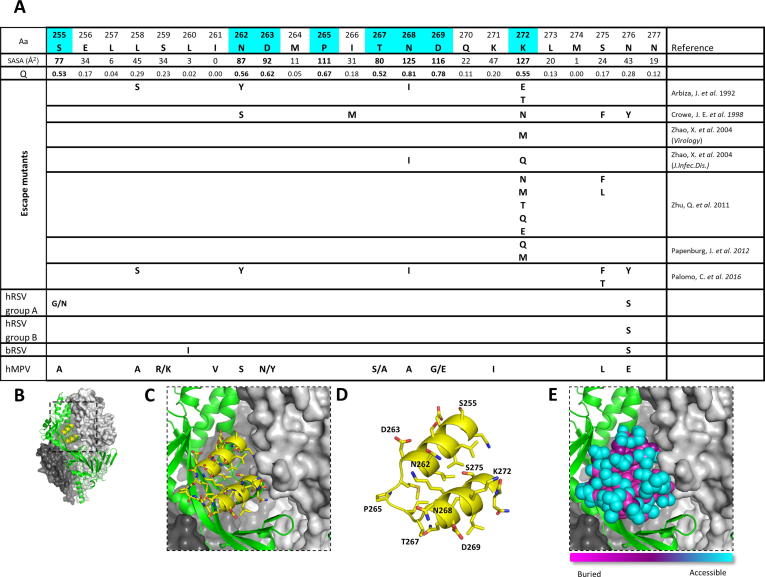
Amino acid changes in antigenic site II. (A) The amino acids included in antigenic site II of hRSV F, A2 strain, are shown in the header of the table with a single letter code. Solvent accessible surface area (SASA) and relative solvent accessibility (Q) values for each residue are shown below this. Cyan indicates highly exposed residues (Q > 0.5) at the prefusion F surface. The amino acid changes selected in escape mutants were reported in the indicated references. The last four lines show the amino acid changes seen when comparing the A2 strain other hRSV group A strain, hRSV group B, bRSV and hMPV F, respectively. (B) Side view of prefusion hRSV F. One protomer is shown as ribbons colored in green with antigenic site II colored in yellow. Molecular surfaces are shown for the other two protomers, colored grey and dark grey. (C) Magnified antigenic site II in yellow. (D) Further magnification with side chains of residues shown as sticks and highly exposed residues labeled. (E) Fraction of solvent accessible surface area for the antigenic site II colored on a per-residue basis: magenta (0%, “buried”) to cyan (100%, “accessible”). (For interpretation of the references to color in this figure legend, the reader is referred to the web version of this article.)

Site II includes mainly two short α-helices connected by a 6 amino acid loop. This structural motif is exposed at the surface of both prefusion and postfusion F (Fig. 2), explaining the capacity of site II specific mAbs to bind to both forms of hRSV F. A number of escape mutants have been isolated by different groups with mAbs that bind to antigenic site II. Most laboratory mutants were selected with mAbs of murine origin [20], [21], [54] but also with Nanobodies [55] or with bovine mAbs [18]. Some of these mutations, specifically those at residues 272 and 275, have been also found in sequences of hRSV F obtained from nasopharyngeal secretions of children infected with hRSV that were treated prophylactically with either palivizumab (Pz) or Mz [25], [26]. In some cases the same mutations were identified in both laboratory mutants and strains found in patients (eg K272M and S275F). All these escape mutants show amino acid changes preferentially at the surface of the prefusion-F (cyan shading in Fig. 7A and E), that allow the avoidance of antibody /nanobody binding. Of note, studies of in vitro escape mutants and of recombinant viruses containing site II mutations isolated in clinical samples have shown that the fitness of these mutants is impaired in comparison with the wild-type RSV in the absence of Pz or Mz [26].

Within the hRSV A group viruses, only two residues (255 and 276) are mutated within antigenic site II; none of the exact amino acid substitutions are shared with an escape mutant. The sequences from the hRSV B strains show full conservation within this portion of the antigenic site (Fig. 7), except for residue 276, that was also found to be mutated in laboratory strains which acquired resistance to mAbs. Again, in these cases the amino acid variant found in circulating B strains (Ser) differs from that found in the laboratory escape mutants (Tyr). This high degree of conservation of antigenic site II between hRSV group A and B correlates with the high degree of cross-reactivity observed with site II specific mAbs. Similarly, the antigenic site II of bRSV F has only two amino acid changes when compared to hRSV F. One of these (L260I) is not surface exposed, and the other (N276S) is the same as that seen in some group A and B viruses (Fig. 7). This result correlates with the finding that the majority of mAbs raised against bRSV F, including at least 12–14 specific for antigenic site II, cross-react with hRSV F [50], [56]. However, a higher number of sites of amino acid changes in hMPV F antigenic site II (12 out of 23 residues), including some found in escape mutants (Fig. 7), likely preclude site II cross-reactivity between hRSV F and hMPV F.

In summary, single amino acid changes, as seen in escape mutants, can have a high impact on antibody binding. The limited variability found in site II across the sequences analysed suggest that site II is not currently under strong enough selective pressure during natural infections to overcome the reduced overall fitness caused by mutations within site II. However, the high variability of this site seen in the circulating hMPV sequences (with a significantly increased probability of mutation), where some of the amino acid variants observed are shared with documented hRSV escape mutants, indicates the potential for this region of the protein to incorporate amino acid changes in a scenario where strong enough selective pressure (eg. in the form of widespread mAb administration) is present.

## 4 Discussion

In the present study, in order to provide insights into the possible effectiveness of differing vaccination/immunisation strategies, we have analysed the variability of RSV F in circulating viruses focusing on F regions including the six major known antigenic sites (Ø and I-V). However, we cannot exclude the possibility that additional sites may also be involved in antibody responses to RSV F; the antigenic sites for a proportion of anti-hRSV F protein human antibodies isolated from adults and infants remain unidentified [13], [42]. Although F regions such as the HRB could be novel, as yet unrecognised, antigenic sites, another alternative is that since all the antibodies used to determine the antigenic sites were isolated using soluble trimeric forms of cleaved F proteins, additional F regions not present in these trimeric forms could also represent relevant antigenic sites for hRSV. Supporting this, the high degree of variability that we observed in p27 in hRSV and bRSV suggests that this region may also constitute an antigenic target that could be under immune pressure in natural infections, possibly in specific age groups: a study found that antibodies to the p27 region were found predominantly in children under the age of 2 [57]. We also found high variability in the SP sequences in hRSV and bRSV. Recent work in HIV has shown that the signal peptide can affect the glycan profile of the adjacent gp120 protein, and thus its immunogenicity [58]; it is possible that a similar process may take place in hRSV.

### Structural implications of F protein variability

The results of previous sections raise important questions about the F glycoprotein from a structural and biological point of view. For instance, why are antigenic sites Ø and V ones that accumulate a high number of amino acid differences between group A and group B hRSV strains? Both sites are located at the apex of the prefusion F protein which includes a series of short α-helices that are rearranged in a long α-helix when the F protein adopts the postfusion conformation (Fig. 2A and B). It may be argued then that the region of prefusion F where antigenic sites Ø and V are placed requires a degree of structural flexibility reflected in a less restricted partial amino acid sequence.

Conversely, the question arises about the relative conservation of antigenic sites III and IV when group A and B strains of hRSV and bRSV are compared. Secondary structural elements of site III and IV are shared between prefusion and postfusion F. However, in the case of site III, they adopt a different spatial arrangement in postfusion F, which explains the higher affinity of site III antibodies to pre- rather than to postfusion F [39]; for site IV antibodies, the affinity remains the same for the two F protein conformations. Site III and site IV are the main regions constituting the inter-protomeric cavity present approximately midway between the apex and tail (C-terminus F1) of the prefusion trimer (Fig. 2A). In addition, site IV is the site around which the short α-helix and the sole parallel strand of the C-terminus F1 unravel and rotate to form the 6-HB motif (Fig. 2B). Thus, antigenic site III (which is also significantly conserved when compared to the hMPV sequences) and IV may be restricted in incorporating sequence changes that would disrupt their essential role in the global structure of the protein, and in the transition from prefusion to postfusion F.

The higher or lower degree of structural restriction in different elements of the F protein may also have immunological consequences, such as the group-dependent neutralisation of hRSV by certain antibodies specific to antigenic site Ø [38].

### Immunological implications of F protein variability

Most viral immunisations used in humans take the form of live attenuated vaccines (measles, mumps, rubella, varicella, rotavirus, polio – oral Sabin, yellow fever, influenza, dengue) or killed virus (influenza, polio, hepatitis A, rabies), and thus stimulate both polyclonal humoral responses and T cell responses to a wide variety of viral proteins. A key question is how viral genetic variability may affect vaccine efficacy. For influenza, antigenic drift, particularly in the haemagglutinin protein, certainly affects the efficacy of seasonal vaccines, requiring regular updating of vaccine strains in order to match circulating strains. However, whether these vaccinations themselves exert selective pressure on circulating viruses remains unclear [59], [60], [61].

However, there are examples where single viral proteins, as opposed to the whole virus, are used as sole components in immunisation campaigns, and these vaccines may be more prone to failure to protect against infection by specific viral subtypes, or to the development of vaccine resistant strains. For example, the human papilloma virus (HPV) vaccine consists of a recombinant viral L1 protein which spontaneously self assembles into a virus like protein, or VLP, and which is used to elicit an antibody response. VLPs are known to stimulate a polyclonal immune response, but in this case the response to HPV L1 protein is subtype specific. Thus, the current vaccines which include genotypes 6, 11, 16 and 18 do not provide protection against subtypes such as 52 and 58, which are more common in Asian countries [62]. Similarly, the current hepatitis B vaccine (HBV) takes the form of recombinant hepatitis B surface antigen (HbsAg) adsorbed onto an adjuvant; the majority of antibodies raised by this vaccine are directed against epitopes located in a single hydrophilic stretch of twenty five amino acids in the middle of the molecule [63]. In the case of the Hepatitis B vaccine, escape mutants with single amino acid changes in the relevant antigen have been identified which evade the immune response in vaccinated individuals [63]. Thus, the HPV and HBV vaccines are two examples in which antibody responses, although polyclonal, only recognise a restricted range of epitopes, determined by the protein used to manufacture the vaccine.

Could hRSV vaccines follow the aforementioned examples of HPV and HBV? The F protein has at least six antigenic sites; the study looking at human antibody responses to the F protein used to characterise antigenic sites Ø to V showed that all the individuals mounted responses to all the antigenic sites [13], and thus none is fully dominant over the others. Therefore, it might be expected that anti-F antibodies elicited by an hRSV vaccine would bind several epitopes, thus reducing the probability of selecting escape mutants that will require the simultaneous incorporation of several independent point mutations. However, it is important to consider that the target population may influence the antibody specificities of the vaccine. Goodwin et al. [42] have recently reported a narrower range of antibody specificities produced by B-lymphocytes obtained from very young children after an hRSV infection than the B-lymphocytes obtained from adults; neutralising responses in adults predominantly targeted site Ø, whilst those in children targeted site III. A thorough analysis of the antibodies induced by future hRSV vaccines will provide information about the necessity of incorporating F proteins from more than one antigenic group if the antibody specificities are skewed to group specific epitopes, such as some of those present in antigenic site Ø. As alluded to previously, type specific (ie hRSV A or B) site Ø antibodies [38] have been documented, laying open the possibility of replacement of certain strains by others if hRSV vaccines were to predominantly induce antibodies to sites containing A or B group specific epitopes.

The argument of restricted antibody repertoire in hRSV vaccines becomes even more relevant in the case of prophylaxis with mAbs, which by definition recognise a single epitope. So far the only mAb used clinically is palivizumab (Pz) which recognizes the site II antigenic site of hRSV F. Pz is recommended for children at high risk of having a severe infection (born preterm or with congenital heart or lung disease) [64] and therefore has limited use. Nonetheless, there are indications of escape mutant viruses with amino acid changes in antigenic site II in a minority of clinical specimens from children treated with Pz but nevertheless infected by hRSV [25], [26], as shown in Fig. 7. A potentially relevant counterpart for RSV is the case of measles. Here, there is little evidence of antigenic drift in circulating populations in response to the widespread administration of live attenuated vaccines, presumably due to the polyclonal response elicited by the whole virus. However, similarly to RSV, escape mutants have been documented in response to monoclonal antibodies in laboratory settings [65]. A clinical example for the selective pressure of neutralising antibodies exists for HIV, where 25% patients treated with the monoclonal antibody ibalizumab showed reduced susceptibility to the agent with prolonged treatment, associated with mutations in the antibody binding site [66]. Taken together, these examples suggest that an immunisation campaign based on the widespread administration of mAbs, rather than vaccines based on a single or multiple RSV proteins, are more likely to lead to selection pressure favouring the emergence of mutants, due to the absence of a polyclonal response to infection.

The study which identified mAb resistant measles strains did not attempt to measure the relative fitness of the mutant strains compared to wildtype. However, for RSV, one of the studies which identified escape mutants in a clinical population [26] found these to have reduced fitness compared to circulating strains. These variants are probably less likely to circulate in the community because of a growth disadvantage. However, as evidenced by the site II variability observed between hRSV and hMPV sequences (Fig. 6), the possibility that an extended passive immunisation programme might lead to the development of alternative variants with improved fitness that could circulate in the community cannot be discarded. In this scenario, the likelihood of an RSV strain emerging in response to the widespread administration of a monoclonal antibody is likely to be determined by the balance between benefits provided to the virus by particular mutations preventing antibody neutralisation, and the consequences to viral fitness of that mutation. This balance, as suggested by the varying proportions of mutations seen in different antigenic sites in our study, is likely to be antigenic site specific: a study examining site Ø escape mutants found no difference in fitness for the mutants [22] compared to circulating strains.

In this respect, the genetic stability of certain antigenic sites, such as sites III and IV, which we find to be conserved to different extents in hRSV A, hRSV B, bRSV and hMPV, may be an advantage in the selection of future mAbs for prophylactic use. In vitro escape mutants have in fact been isolated for site IV antibodies, but information on the fitness of these variants is not available [19]. For site III, the attempt to isolate in vitro escape mutants against MPE8 failed [44], and, in addition, the unique site III escape mutant isolated using a nanobody showed strongly impaired fitness, where viral growth was not observed in the absence of the nanobody [23]. These data support the limited potential of this region to mutate, as reflected in the limited sequence variability of the antigenic site when comparing hRSV and related species, and highlight its potential use as a target for immunisation programs.

## 5 Conclusion

If vaccination or other prophylactic measures take approaches that could place selective pressure on particular F protein epitopes, our analyses show that a considerable degree of amino acid variability is tolerated in hRSV F and closely related viruses. It would therefore seem prudent to coordinate efforts for prospective sample collection that might identify potential evolutionary changes in the virus driven by these immunisation programs.

## ICMJE statement

Our submission was delayed by the untimely and unexpected death of the lead author for this paper, Professor Jose Antonio Melero. The paper was at an advanced stage when Professor Melero died. He therefore meets authorship criteria apart from consent to the final version - but we believe he would have consented given no major changes have been made to the conclusions of our paper since his last input. In these circumstances, all authors, except for Professor Melero, attest they meet the ICMJE criteria for authorship.

## Statement of financial support

T.C. Williams is the recipient of a Wellcome Trust Award [204802/Z/16/Z]. H. Nair and H. Campbell are members of the Respiratory Syncytial Virus Consortium in Europe (RESCEU). RESCEU has received funding from the Innovative Medicines Initiative 2 Joint Undertaking under grant agreement No. 116019. This Joint Undertaking receives support from the European Union’s Horizon 2020 research and innovation programme and EFPIA. This work also aligns with the research of the RESPIRE Unit which was commissioned by the National Institute of Health Research using Official Development Assistance (ODA) funding.
