# Supplementary material for: Antigenic and sequence variability of the human respiratory syncytial virus F glycoprotein compared to related viruses in a comprehensive dataset
Source: Vaccine. 2018 Oct 29;36(45):6660–73. doi: 10.1016/j.vaccine.2018.09.056 (PMC6203811; doi:10.1016/j.vaccine.2018.09.056)
Supplement: Supplementary data 1 [file mmc1.pptx]

## Slide 1
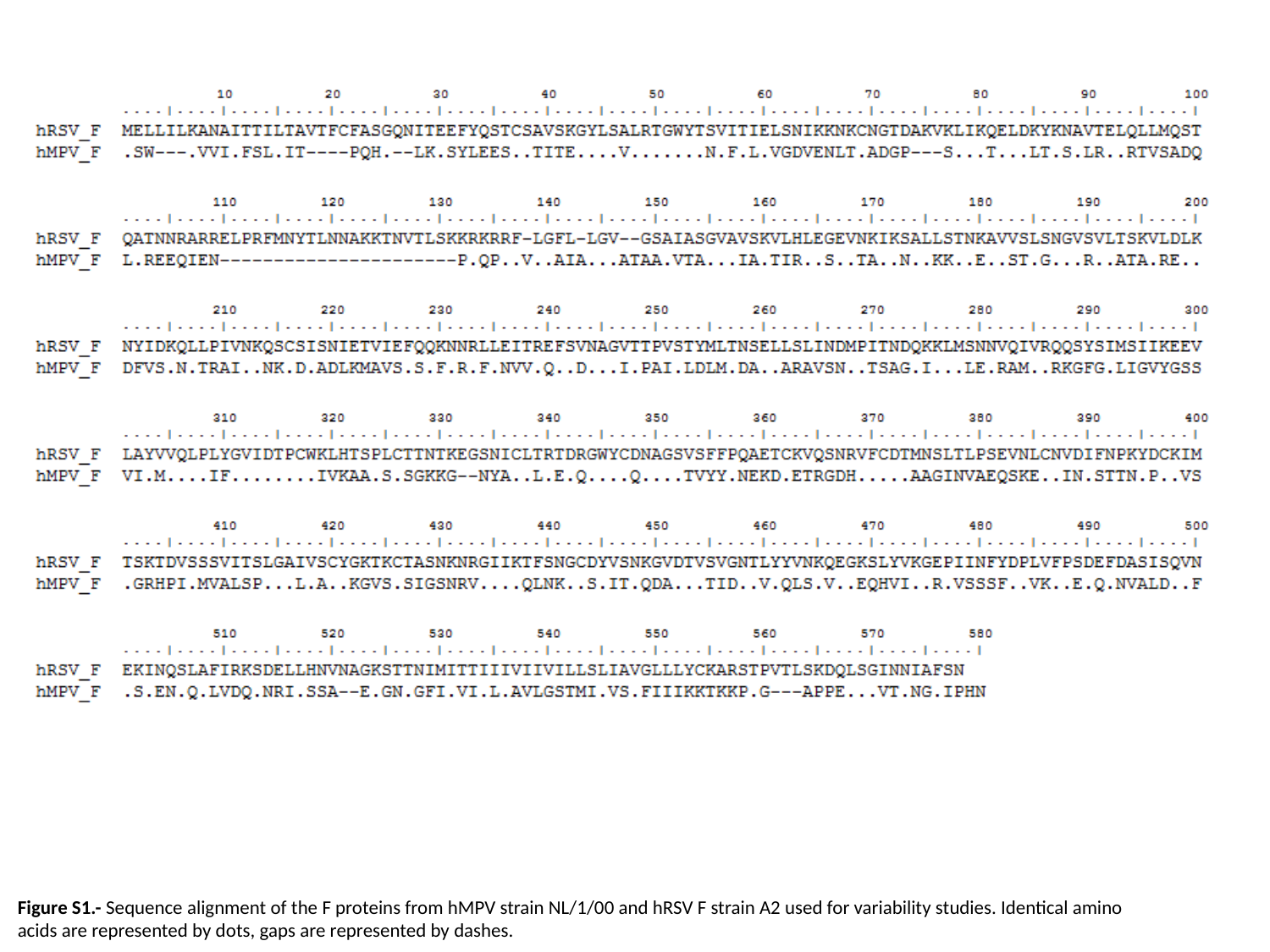

Figure S1.- Sequence alignment of the F proteins from hMPV strain NL/1/00 and hRSV F strain A2 used for variability studies. Identical amino acids are represented by dots, gaps are represented by dashes.

## Slide 2
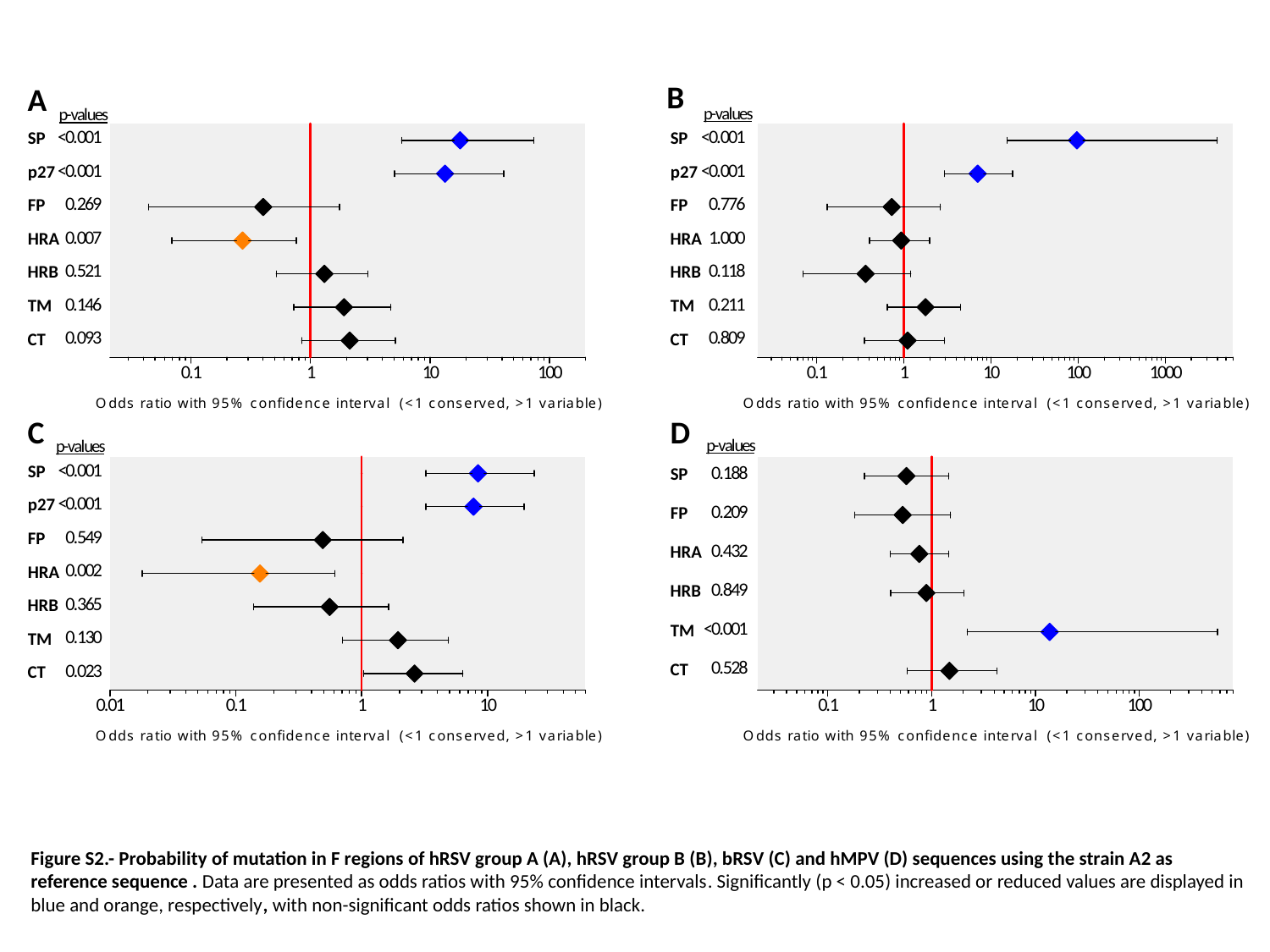

B
A
SP
p27
FP
HRA
HRB
TM
CT
SP
p27
FP
HRA
HRB
TM
CT
C
SP
p27
FP
HRA
HRB
TM
CT
D
SP
FP
HRA
HRB
TM
CT
Figure S2.- Probability of mutation in F regions of hRSV group A (A), hRSV group B (B), bRSV (C) and hMPV (D) sequences using the strain A2 as reference sequence . Data are presented as odds ratios with 95% confidence intervals. Significantly (p < 0.05) increased or reduced values are displayed in blue and orange, respectively, with non-significant odds ratios shown in black.

## Slide 3
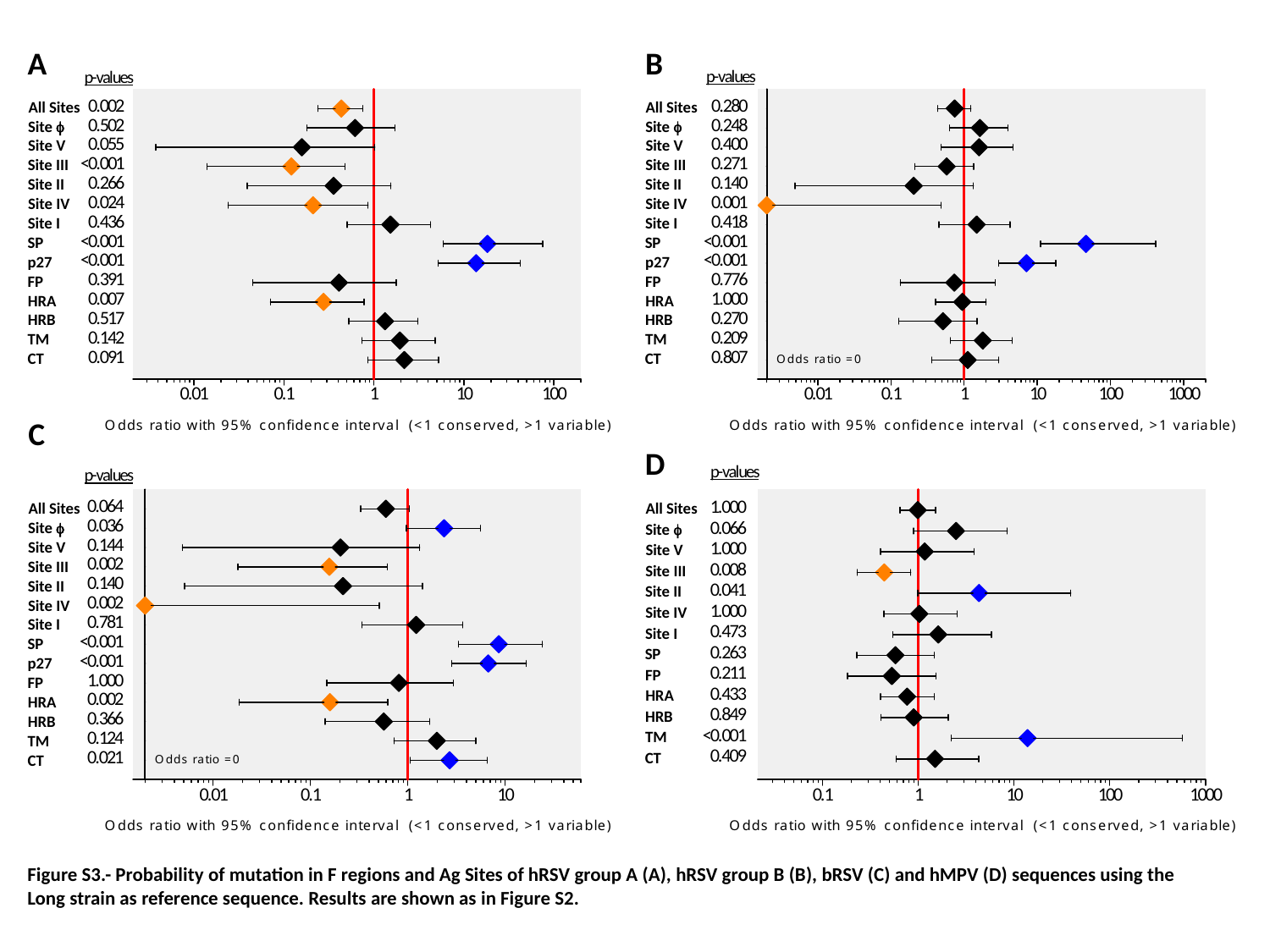

A
B
All Sites
Site 
Site V
Site III
Site II
Site IV
Site I
SP
p27
FP
HRA
HRB
TM
CT
All Sites
Site 
Site V
Site III
Site II
Site IV
Site I
SP
p27
FP
HRA
HRB
TM
CT
C
D
All Sites
Site 
Site V
Site III
Site II
Site IV
Site I
SP
p27
FP
HRA
HRB
TM
CT
All Sites
Site 
Site V
Site III
Site II
Site IV
Site I
SP
FP
HRA
HRB
TM
CT
Figure S3.- Probability of mutation in F regions and Ag Sites of hRSV group A (A), hRSV group B (B), bRSV (C) and hMPV (D) sequences using the Long strain as reference sequence. Results are shown as in Figure S2.

## Slide 4
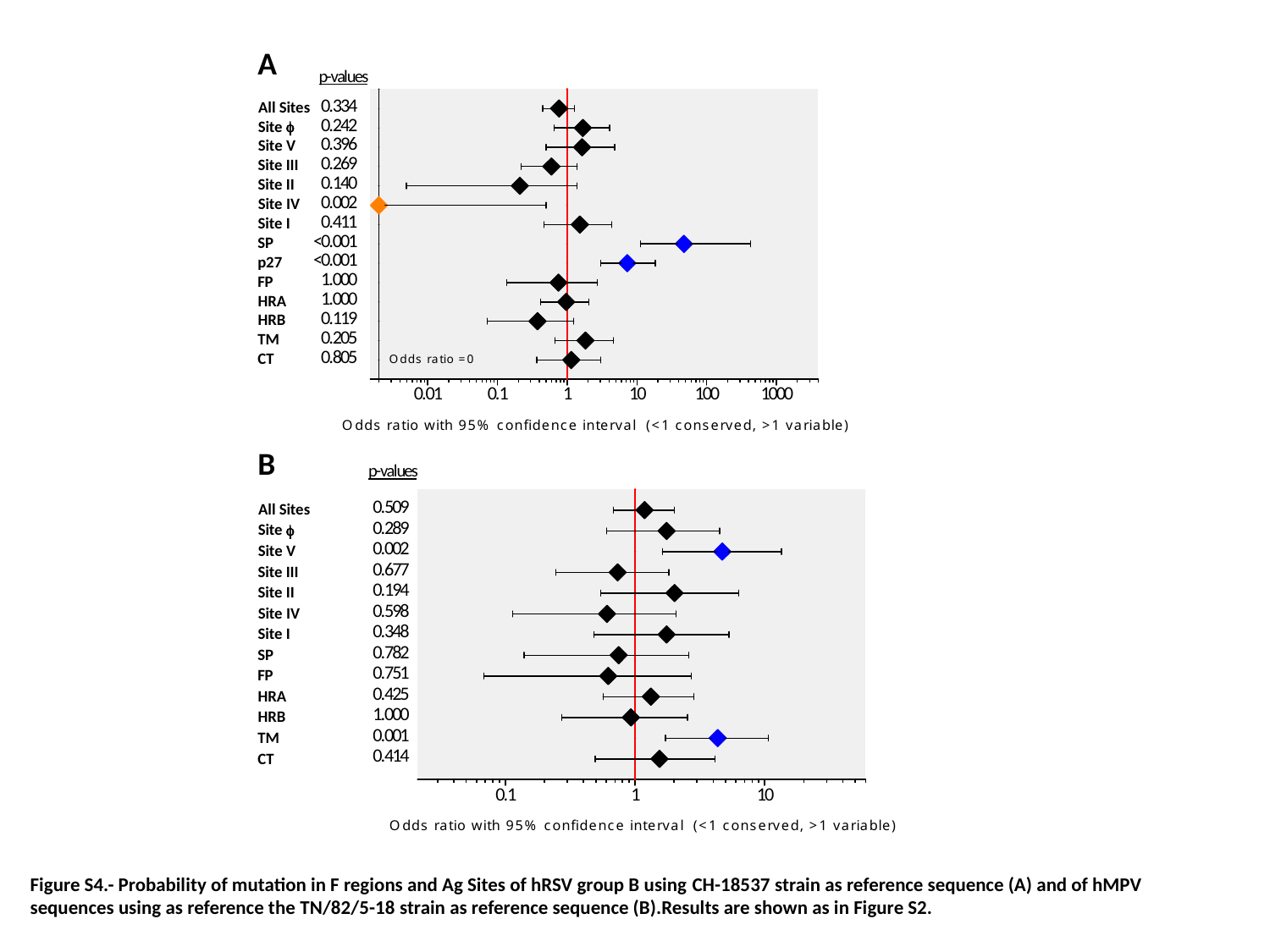

A
All Sites
Site 
Site V
Site III
Site II
Site IV
Site I
SP
p27
FP
HRA
HRB
TM
CT
B
All Sites
Site 
Site V
Site III
Site II
Site IV
Site I
SP
FP
HRA
HRB
TM
CT
Figure S4.- Probability of mutation in F regions and Ag Sites of hRSV group B using CH-18537 strain as reference sequence (A) and of hMPV sequences using as reference the TN/82/5-18 strain as reference sequence (B).Results are shown as in Figure S2.

## Slide 5
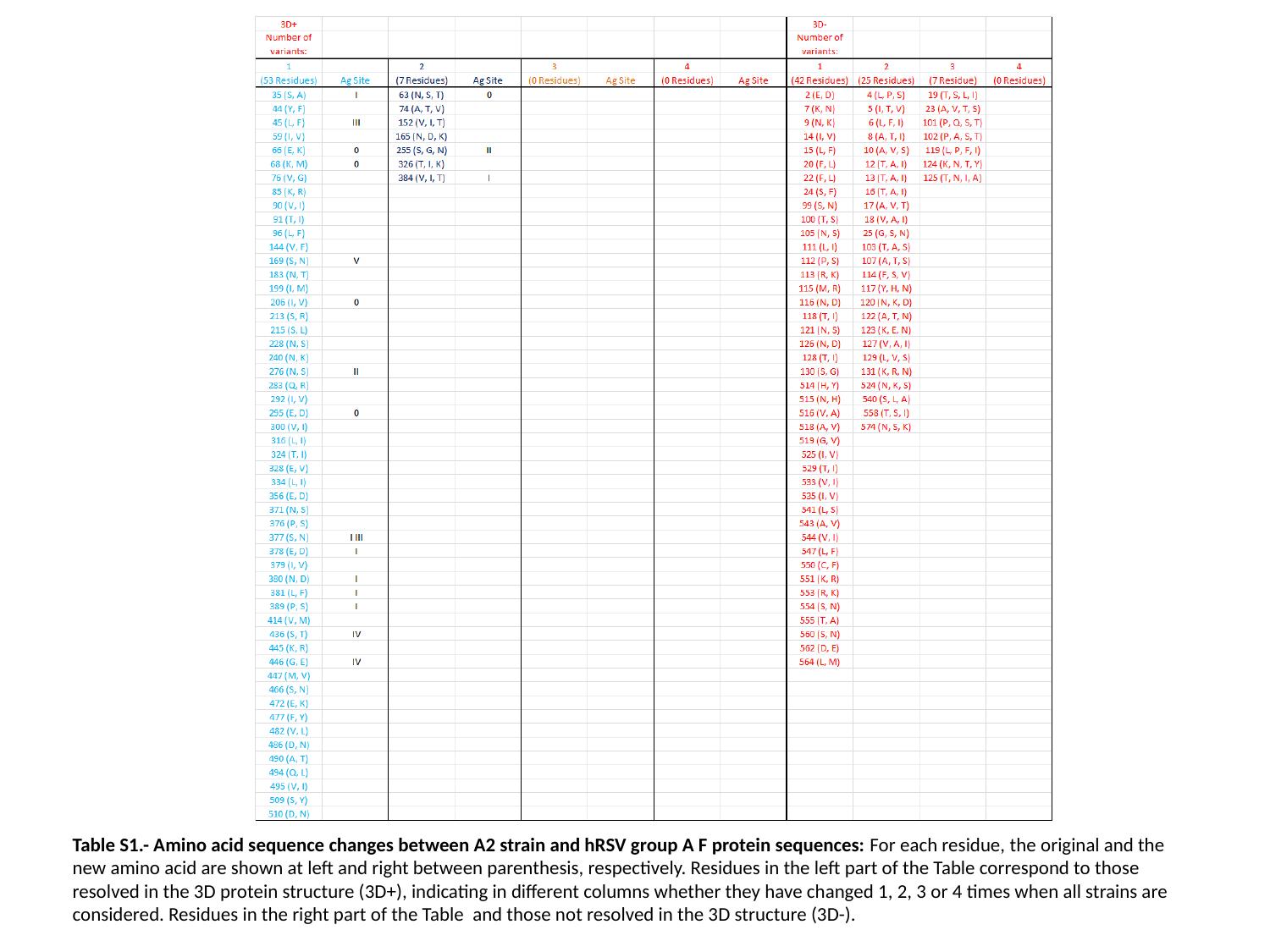

Table S1.- Amino acid sequence changes between A2 strain and hRSV group A F protein sequences: For each residue, the original and the new amino acid are shown at left and right between parenthesis, respectively. Residues in the left part of the Table correspond to those resolved in the 3D protein structure (3D+), indicating in different columns whether they have changed 1, 2, 3 or 4 times when all strains are considered. Residues in the right part of the Table and those not resolved in the 3D structure (3D-).

## Slide 6
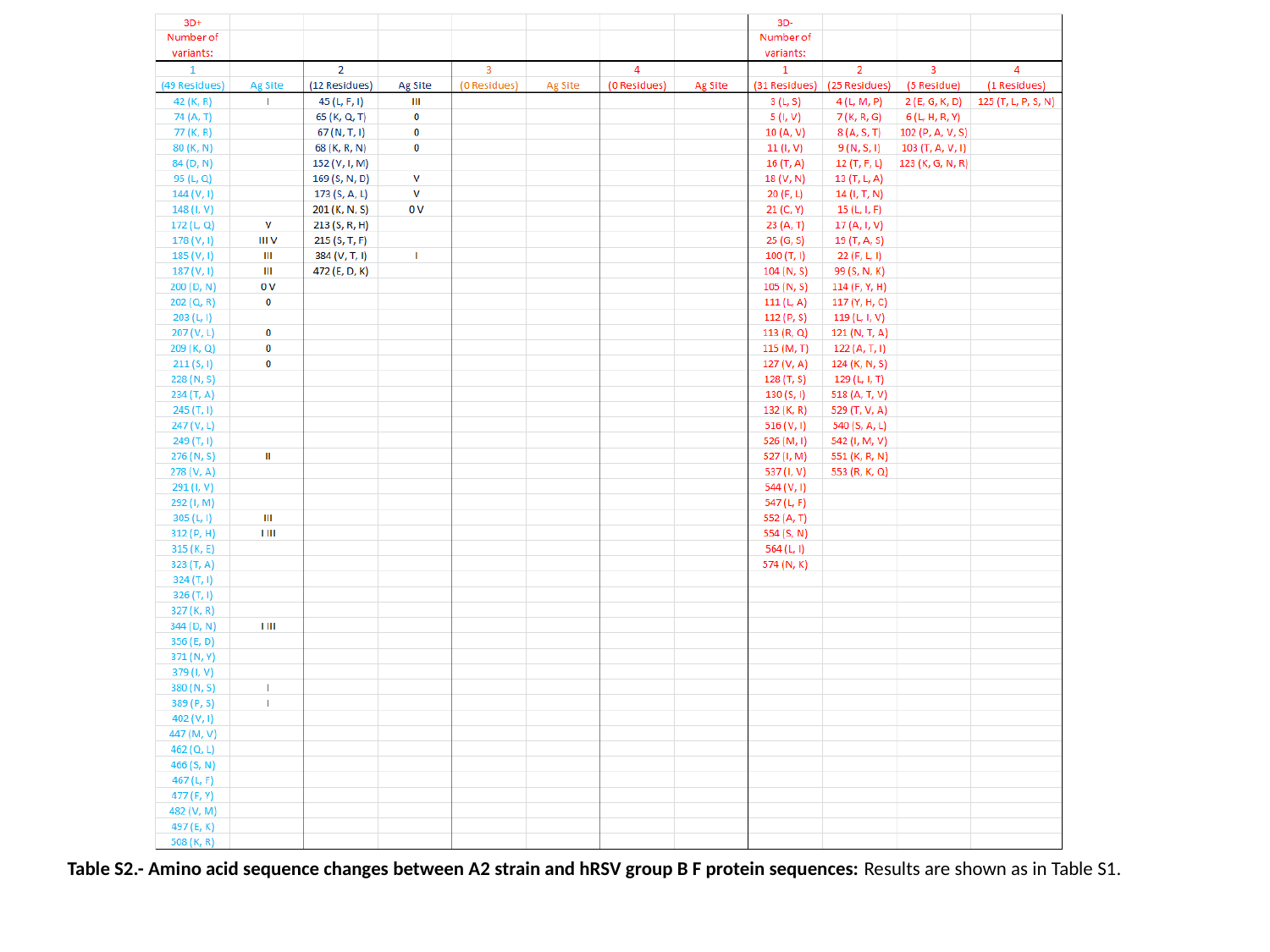

Table S2.- Amino acid sequence changes between A2 strain and hRSV group B F protein sequences: Results are shown as in Table S1.

## Slide 7
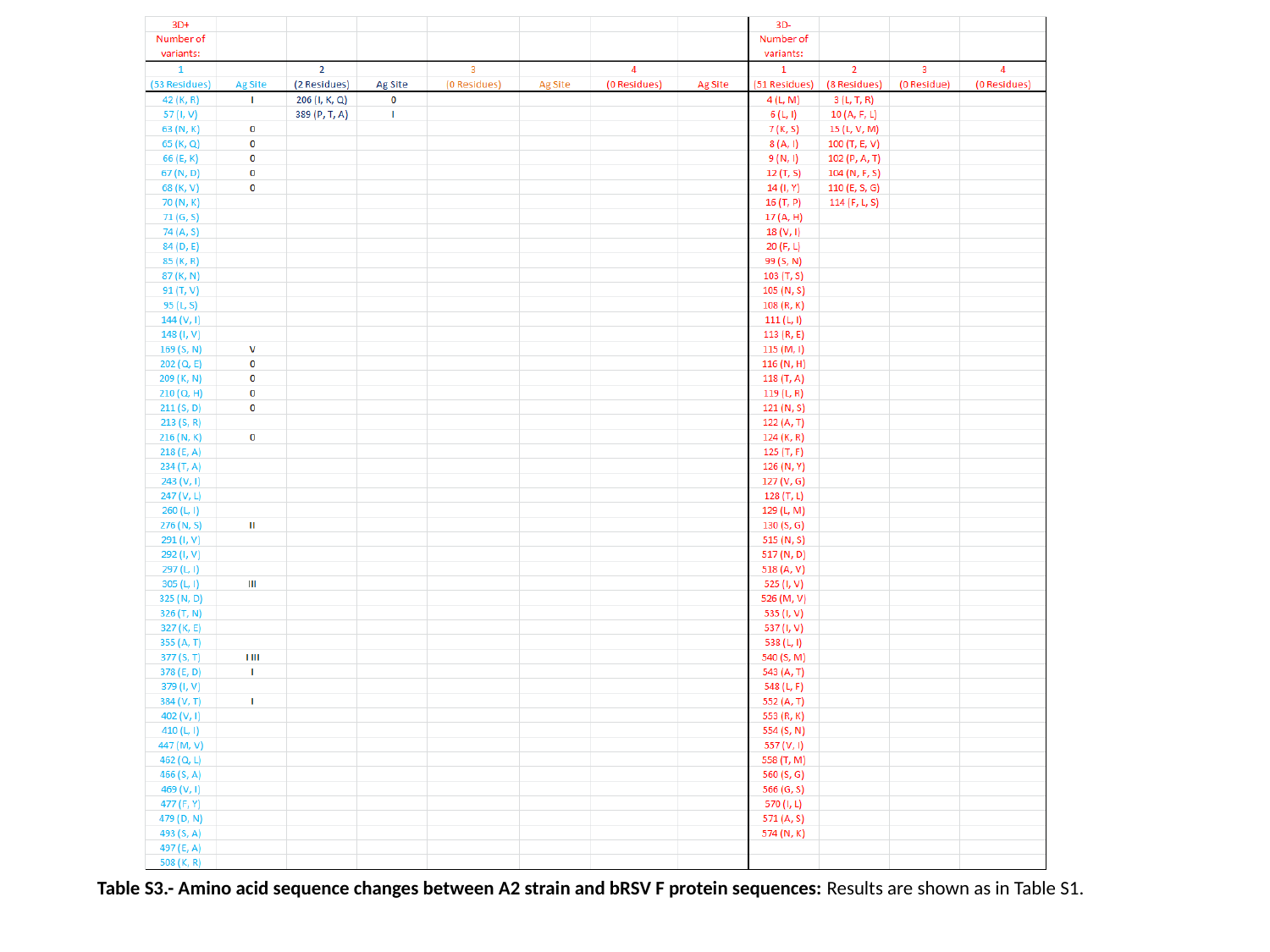

Table S3.- Amino acid sequence changes between A2 strain and bRSV F protein sequences: Results are shown as in Table S1.

## Slide 8
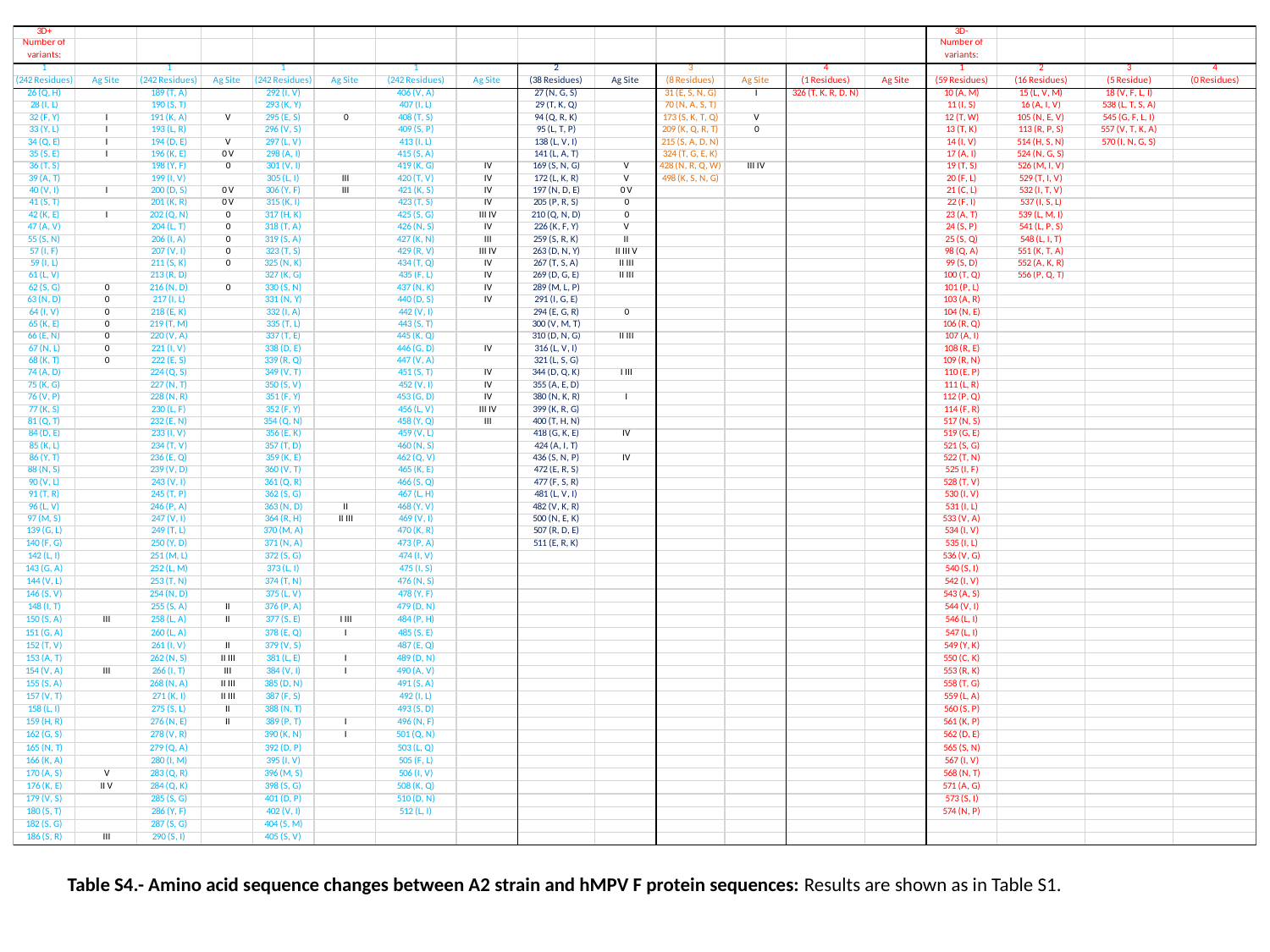

Table S4.- Amino acid sequence changes between A2 strain and hMPV F protein sequences: Results are shown as in Table S1.
